# Supplementary material for: Identification of photoperiod-induced specific miRNAs in the adrenal glands of Sunite sheep (Ovis aries)
Source: Front Vet Sci. 2022 Jul 22;9:888207. doi: 10.3389/fvets.2022.888207 (PMC9354845; doi:10.3389/fvets.2022.888207)
Supplement: Supplementary file 1 [file Data_Sheet_1.ZIP › Supplementary file/miRDeep2 file.zip!/miRDeep2 file/Know_Structure/SP42A2/oar-mir-543.pdf]

miRBase precursor : oar-mir-543
Total read count : 18693
oar-miR-543-5p read count : 6
oar-miR-543-3p read count : 18641
remaining reads : 46

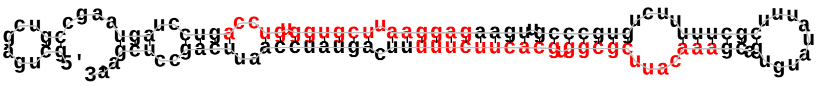

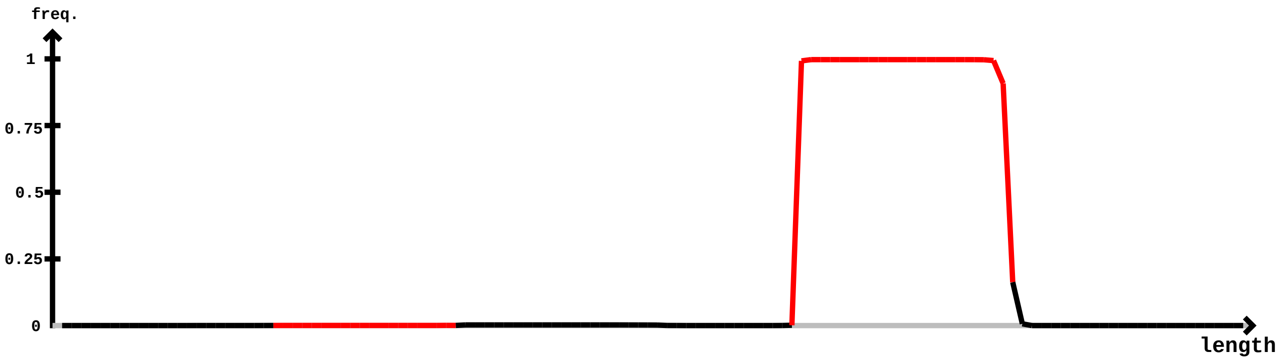

oar-miR-543-3p

oar-miR-543-5p

```
5'- gcugagcugccgaaugauccugaccuguggugcuuaaggagaaguugcccgugucuuuuucgcuuuauaugugacgaaacauucgcggugcacuucuuuuucaguauccaauucagccucgaa  -3'   exp
    ((......)).....((..((((..((.((((((.((((((((((.(((((((....((((((.........).)))))....))))).)))))))))))).))))))))..))))..))...   reads   mm
    .............augauccugaccuguggugcuuaagga...................................................................................   1       0
    ..................ccugaccuguggugcuuaagga...................................................................................   1       0
    ....................ugaccuguggugcuuaagga...................................................................................   2       0
    .....................gaccuguggugcuuaagga...................................................................................   2       0
    ......................accuguggugcuuaagga...................................................................................   1       0
    ......................accuguggugcuuaaggag..................................................................................   1       0
    .......................................agaaguugcccgugucuuu.................................................................   1       0
    .......................................agaaguugcccgugucuuuu................................................................   2       0
    .......................................agaaguugcccgugucuuuuu...............................................................   1       0
    .......................................agaaguugcccgugucuuuuuc..............................................................   2       0
    ........................................gaaguugcccgugucuuuuuc..............................................................   1       0
    ........................................gaaguugcccgugucuuuuucg.............................................................   2       0
    .........................................aaguugcccgugucuuuuuc..............................................................   1       0
    .........................................aaguugcccgugucuuuuucg.............................................................   28      0
    .........................................aaguugcccgugucuuuuucgc............................................................   3       0
    .........................................aaguugcccgugucuuuuucgcu...........................................................   3       0
    ..........................................................................cgaaacauucgcggugcacuucu..........................   3       0
    ...........................................................................gaaacauucgcggugcacuu............................   2       0
    ...........................................................................gaaacauucgcggugcacuucu..........................   16      0
    ...........................................................................gaaacauucgcggugcacuucuuu........................   2       0
    ............................................................................aaacauucgcggugcac..............................   1       0
    ............................................................................aaacauucgcggugcacu.............................   2       0
    ............................................................................aaacauucgcggugcacuu............................   5       0
    ............................................................................aaacauucgcggugcacuuc...........................   56      0
    ............................................................................aaacauucgcggugcacuucu..........................   1575    0
    ............................................................................aaacauucgcggugcacuucuu.........................   13883   0
    ............................................................................aaacauucgcggugcacuucuuu........................   2886    0
    ............................................................................aaacauucgcggugcacuucuuuu.......................   121     0
    .............................................................................aacauucgcggugcacuucu..........................   7       0
    .............................................................................aacauucgcggugcacuucuu.........................   52      0
    .............................................................................aacauucgcggugcacuucuuu........................   27      0
    .............................................................................aacauucgcggugcacuucuuuu.......................   2       0
    ..............................................................................acauucgcggugcacuucuuu........................   1       0
```
